# Perceived and Preferred Social Support in Patients Experiencing Weight Regain After Bariatric Surgery—a Qualitative Study

**DOI:** 10.1007/s11695-020-05128-5

**Published:** 2020-11-17

**Authors:** Liisa Tolvanen, Åsa Svensson, Erik Hemmingsson, Anne Christenson, Ylva Trolle Lagerros

**Affiliations:** 1 Clinical Epidemiology Division, Department of Medicine Solna, Karolinska Institutet, SE-171 76 Stockholm, Sweden; 2 Center for Obesity, Academic Specialist Center, Stockholm, Sweden; 3 Department of Health Sciences, Mid Sweden University, Östersund, Sweden; 4 Åstrand Laboratory of Work Physiology, The Swedish School of Sports and Health Sciences, Stockholm, Sweden

**Keywords:** Interview, Metabolic surgery, Obesity, Support, Thematic analysis, Weight regain

## Abstract

**Purpose:**

While bariatric surgery generally shows successful weight loss outcomes in patients with obesity, weight regain exists. The aim of this qualitative study was to improve understanding of how patients with substantial weight regain after bariatric surgery experienced the support from family, friends, and healthcare providers, and what kind of support they had preferred.

**Materials and Methods:**

Qualitative data were collected from semi-structured interviews with 16 participants. Mean weight regain from surgery to interview was 36%. The transcribed interviews were analyzed with thematic analysis.

**Results:**

Two main themes and seven sub-themes were formulated. The theme, *A lonely struggle*, illustrates patients’ feelings of abandonment and struggle during weight regain due to lack of support or unfavorable treatment. Participants commonly blamed themselves for re-gaining weight, and shame made them reluctant to engage in social activities or seek medical care. The theme, *Others as sources of compassion and control*, covers what support they desired, as well as had perceived to be helpful. Exercising or eating healthy with others was appreciated and felt supportive. Pro-active healthcare support and access to dietitians, physiotherapists, and psychological support were desired.

**Conclusion:**

To optimize the effect of bariatric surgery, support may need to be individualized and lifelong. Since shame and self-blame in patients with weight regain may hinder seeking professional help, care providers may need to initiate follow-up visits. Empathetic and non-judgmental support, access to multidisciplinary healthcare team, as well as peer-support groups may be beneficial to counteract weight regain post-bariatric surgery.

## Introduction

Although bariatric surgery is an effective method for weight loss, less successful outcomes have been reported, including substantial weight regain. Apart from recurrence of medical risks, weight regain may also have a negative impact on the quality of life (QoL) [1]. One and a half to 2 years post-surgery, most patients have either stabilized their weight, or in some cases regained some weight [2]. The exact prevalence of weight regain is unclear, both due to low follow-up rates [3] and to large variations in how weight regain is defined [4]. Weight regain seems to be especially common after gastric banding, but may occur regardless of surgical method [5].

Many complex factors may contribute to the phenomenon of weight regain [6]. Difficulties in maintaining healthy eating and exercise habits seem to be of importance [6, 7]. Bariatric surgery affects gut hormone levels and facilitate weight loss through increased satiety, resulting in a reduced intake of food [8]. However, the effect on hunger and satiety hormones vary between individuals, and over time, which may contribute to weight regain if appetite and portion sizes increases [8, 9]. For some, reactive hypoglycemia may contribute to increased grazing or snacking behavior which in turn may support weight regain [9, 10], while for others lack of support and missed follow-up visits may be contributing factors leading to weight regain [7].

It is important to identify factors that could improve post-bariatric care, in order to prevent or limit weight regain. Therefore, we aimed to explore how patients who had undergone bariatric surgery and had regained more than 10% weight from the lowest weight since bariatric surgery experienced support from family, friends, and healthcare providers, as well as what kind of support they believed could have minimized weight gain.

## Materials

### Study Participants and Recruitment

Purposeful sampling [11] was used to recruit patients from a publicly funded obesity specialist clinic. Participants were ≥ 18 years, Swedish-speaking, with BMI ≥ 35 kg/m^2^ (after weight regain) and with ≥ 10% weight regain after gastric bypass or sleeve gastrectomy. Persons with weight regain after gastric banding were not included, since weight regain is common after this procedure; additionally the method is no longer performed in Sweden [12]. However, persons who had undergone gastric banding and then had gastric bypass or sleeve gastrectomy were eligible for the study.

Participants with an ongoing patient relation with the first author (LT) were excluded, since they were considered to be in a dependent position. Of 19 patients asked, 16 were included. Persons who declined participation were all females. They did not differ substantially to included participants regarding body mass index and amount of regained weight, but they were on average slightly younger. It was emphasized that participation was voluntary, and there was no incentive to participate. Before the interview, participants received oral and written study information, and written informed consent was obtained.

Though the collected data were deemed rich and sufficient after thirteen interviews, three more interviews were conducted to ensure that informational redundancy [11, 13] was attained, as well as to increase the sample variation in terms of gender, country of origin, and age [11]. Differences and similarities in the data material were continuously discussed. After sixteen interviews, a joint decision was made that the data material was sufficiently informative and that further interviews would be redundant [11, 14].

## Method

### Design of the Study

A qualitative design with in-depth individual semi-structured interviews to explore perceptions, feelings, and opinions about postoperative support was chosen. The inductive approach enabled generation of new insights and understanding from the material [11].

### Data Collection

Individual interviews were conducted from April 2018 to December 2019. A semi-structured interview guide was used to ensure that all the areas (experiences of weight regain, and post-surgery support from healthcare professionals, family, and friends) were covered. Background characteristics such as age, surgery date, and weight development after surgery were collected at the beginning of each interview. Interviews lasted 32–79 min (mean 60 min), and were recorded digitally. Interviews were transcribed verbatim, and participants were offered a printed copy of the interview text to check for misunderstandings. No changes were requested.

### Analysis

**Fig. 1:**
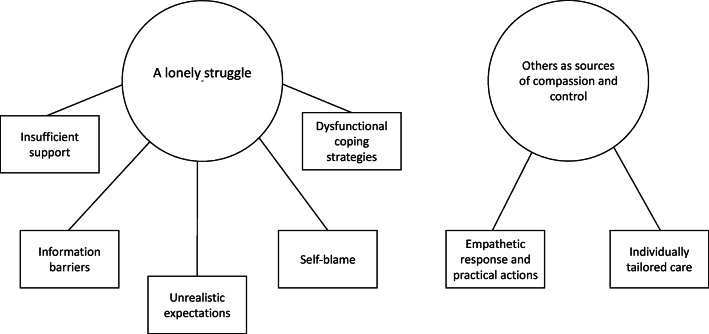
Thematic map with two main themes and seven sub-themes

Thematic analysis [15] was used to assess the latent content of the data and followed six steps. Step 1: Notes were made during data collection and the interview transcripts were read several times to get an overall impression of the corpus. Step 2: Data extracts were identified and labeled with a code, describing the essence of the content. Step 3: Initial codes were grouped manually and compared with each other regarding similarities and differences, and formed preliminary themes (Table 1). Step 4: The themes were reviewed, merged, and renamed until agreement was reached. Step 5: The themes were “defined and refined” [15], i.e., final decisions were made about how themes were called and presented. Throughout the subsequent steps, interview texts were revisited, and thematic maps used to allow us to visualize, discuss, and review overarching themes and their interrelationships. A final thematic map (Fig. 1) illustrated how themes and sub-themes were connected. Step 6: Quotes were chosen to illustrate how themes remained grounded in participants’ descriptions.

**Table 1:** Example of the analysis process with coded data extracts under preliminary themes

Data extract	Initial codes	Sub- Themes (preliminary)	Themes (preliminary)
“I feel like I’ve been referred back and forth, because I want help to lose weight, but nobody has provided proper help.”	Nobody’s responsibility	Lack of support and follow-up	Insufficient support
“It felt good [that my sister accompanied me here]. And I hugged her, and I said thank you for coming along today.”	Company during health care visits	Concrete actions	Beneficial support

To reduce risk for researcher bias (e.g., pre-conceptions, attitudes, and interaction with participants) [11], and to provide additional rigor and reflexivity to the findings, researcher triangulation was performed [11]. The first author (LT), with experience from specialist obesity care, conducted and transcribed the interviews, and commenced the analysis. The second to last author (AC), with experience from specialist obesity care and qualitative research, also conducted the same initial analysis. Thereafter, they discussed the suggestions of codes and themes. Notes were compared to ensure that all relevant passages were assessed, and codes were agreed on. As part of the self-reflexivity process, the authors (LT, AC) separately wrote down their ideas and reflections on the material continuously. The co-authors added interdisciplinary critique.

## Results

### Participants Characteristics

Participants (*n* = 16) were mostly female (*n* = 12), middle-aged (mean age 49 years) with a mean weight regain of 36% from the lowest weight since last bariatric surgery (Table 2). All participants had undergone gastric bypass in Sweden between 2004 and 2016. For three of the participants, gastric bypass was a re-operation (gastric banding, *n* = 2 and sleeve gastrectomy, *n* = 1). Most participants were Nordic born (*n* = 11), and five (31%) were from the Middle East, Asia, or South America. Most patients (*n* = 13) were employed or studied. Some had various kinds of income support. Several participants described problems with depression, pain, addiction, and other illnesses, either through-out life or post-surgery. Marital status varied, and most patients (*n* = 12) had children.

**Table 2:** Characteristics of participants (*n* = 16)

Characteristics	Value Mean (range) unless otherwise stated
Gender	
Women (*n*) (%)	12 (75)
Age (year)	
Mean (range)	49 (20–64)
Origin	
Nordic origin (*n*) (%)	11 (69)
Type of bariatric surgery^a^	
Gastric bypass (n) (%)	16 (100)
Time since last^a^ bariatric surgery to interview (y)	
Mean (range)	10 (3–15)
Weight loss (%)^b^	
Total weight loss at the lowest weight since last^a^ bariatric surgery	35 (14–50)
Weight regain (%)^b^	
Postoperative weight regain from the lowest weight since last^a^ bariatric surgery	36 (12–71)
Body weight	
Body weight, pre-operative (kg)^b^	146 (96–205)
Highest body weight after weight regain (kg)^b^	128 (80–177)
Body mass index (BMI)	
Body mass index, BMI, preoperative (kg/m^2^)^b^	52 (42–70)
Highest BMI after weight regain (kg/m^2^)^b^	46 (36–66)

^a^For three of the participants gastric bypass was a re-operation, for two after gastric banding, and for one after sleeve gastrectomy

^b^In some cases weight and height were self-reported

### Themes

Two main themes and seven sub-themes (Fig. 1) were formulated. The number after each quote refers to which participant provided the statement, and “//” indicates that irrelevant text has been removed.

#### Theme 1—a Lonely Struggle

##### Insufficient Support

Though most participants had attended follow-up visits after surgery, all but one patient had experienced the support as insufficient or lacking important components. Some perceived the support from healthcare as too sparse during the first year, while others felt it was too short a period, as difficulties in weight management occurred later on.

Then I lacked support to continue to adhere to the proposed diet, the size of the portions that I should and that was ah... There I needed support and I think it worked badly, the follow-up. No. 5

Participants felt responsible for initiating contact with healthcare, but were uncertain where they could turn for support. Some were referred back and forth between hospitals and primary care with insufficient communication between medical institutions.

Nobody gave me anything, no one cared and I was angry and disappointed and ‘I just can’t be bothered’… No. 12

Participants explained that lack of support increased their feelings of shame, sadness, and abandonment. The attended follow-up visits were perceived as too focused on weight and measurements, and too little on diet, psychological support, or motivational support. Healthcare staff did not bring up the topics of eating disorders or addiction-related problems, and some described the caregiver as uninterested and unhelpful.

I would say the support were merely information about facts. In my opinion one needs a psychologist // Like once or twice a month to start with // because right now it has been nothing but follow-up of weight, BMI, fat percent… No. 16

The support from friends and family varied, but was described by many participants as not only insufficient, but sometimes even stigmatizing or discouraging. Participants felt misunderstood and were sometimes the target for negative remarks like nagging and hints about their eating and weight, that they felt were impossible to ignore.

She [mother in law] constantly comments on my weight, “you should lose weight”, and so I don’t feel like answering her anymore. Losing weight is the only thing I think about, still, she keeps commenting. No. 2

##### Information Barriers

Though most participants described they received preoperative surgery and diet information, some had found the information difficult to understand, or had forgotten it. One participant found written information unhelpful, since information leaflets were lost or remained unread. Several participants noted they were not receptive to information about weight regain or risks, while being focused on losing weight. Furthermore, dietary advice was sometimes perceived as unreliable, and information varied between bariatric surgery clinics.

…and the information from different health care clinics varied a lot, so you made sure to compare what they said //…and took the information you got and verified it [the information] with the online forum. No. 15

##### Unrealistic Expectations

Many participants relied on the effect of surgery to achieve a large and permanent weight loss and were unprepared for having to continue the struggle with their weight, like before surgery.

I might have relied too much on the method itself and did not realize what I need to do myself. It’s not just a quick fix. No. 2

Participants reported being unaware of the possibility of poor weight loss and weight regain, and of the importance to follow dietary guidelines. Effortlessly losing weight in the beginning gave one participant the impression that she did not need to work actively for her weight loss, and when weight regain started, it was difficult to adjust the diet. Some were disappointed that the procedure itself did not stop them from eating large portions and unhealthy food.

…so I am disappointed. I feel betrayed. I feel that health care has deceived me. Because when I came home from the hospital after having had the surgery, I was so happy. // I was crying, thinking ‘finally, now I will be thin.’ No. 12

##### Self-Blame

Participants felt responsible to resist temptations by themselves. They blamed themselves for not following dietary advice, not having worked hard enough to prevent weight regain, or noticing it in time, and not having asked for help soon enough.

…it is my own fault and yes I know that, and I am a bad person and I should be more controlled and more disciplined and less lazy and… No. 5

Weight regain induced feelings of shame and guilt, which acted as a barrier for seeking help.

And it was like… I felt ashamed… that I had failed…that I kept gaining weight but could not do that much about it. No. 3

Participants commonly tried to hide unhealthy eating habits, weight regain, and negative feelings from their family and friends. Several expressed hopelessness and despair, thinking there might be nothing more to do.

I thought, now I have done this surgery, I've had the world's chance, yet I regain weight. I must be stupid. No. 8

##### Dysfunctional Coping Strategies

Participants reported that weight regain induced old thought patterns with dysfunctional dietary coping strategies, including disordered eating.

I know a girl who does it [vomits]. It helps her. // I may have to try sometime if I am not feeling good. It may be much better to do that than to lay down // Obviously, it’s better to throw up than lay down and absorb calories. No. 3

Negative emotions induced emotional eating, which hampered weight management. Three participants described hazardous alcohol use.

You are hungry and need to eat, but you cannot, you cannot get anything down. It is so easy to end up in other addictions and stuff. Then, for me it was alcohol. Drinking was the easy way. No. 11

The fear of stigmatizing treatment was described as a barrier for activities that included social contacts. One patient avoided swimming, yet another stayed indoors because other people commented on her body size. Even participating in an obesity treatment group was avoided for shame of being perceived as the heaviest one. Hiding at home, social isolation, and avoiding healthcare meetings were the strategies that provided relief from shame, while obstructing any possibility to receive social support.

I did not dare to go out [because of comments].//I was really sad and depressed. To avoid that, I stayed at home. I stayed at home and did not want to go out for 2-3 years. No. 6

#### Theme 2—Others as Sources of Compassion and Control

Though data about insufficient support were plentiful, participants also gave examples of how specific social support empowered them and affected their psychosocial well-being.

##### Empathetic Response and Practical Actions

Empathetic and non-judgmental approach, shared experiences, and pro-active healthcare increased participants’ perceived self-efficacy, motivation, and hope. Examples are as follows: initiating walks, exercising, eating healthy food together, or accompanying participants to healthcare visits, as well as supporting participants in refraining from hazardous consumption of alcohol.

My husband, too, like, he says, ‘Let’s do it together.’ Walk, or do something else. Or occasionally I have tried to cook, I have not fried or deep-fried, just more like boiled or made a salad or like lighter dishes and then he eats with me too. No. 4

One patient found that having her bowel and gastric volume examined and receiving the surgeons’ confirmation that nothing was wrong, positively inspired her to continue searching for lifestyle support. Receiving weight loss medication, clear information, and diet advice, as well as being referred to a dietitian or psychologist was also appreciated.

When healthcare professionals, family members, or friends gave positive comments, comforted them, showed respect, love, acceptance, and were perceived as understanding, participants felt better and more self-confident.

It’s when they think that you are good even if you are... like my boyfriend, now when I am eating according to LCHF-diet [low carbohydrate-high fat-diet] and I can’t see any results on the scale, and then he says, ‘no, but it can take a while before your body understands that something is happening.’ That is great. // He can see the bigger picture. No. 8

Several participants expressed a desire to go through re-operation. Furthermore, participants expressed that it had been meaningful to share experiences with other people who had undergone bariatric surgery. Such encounters were enabled via online forums.

##### Individually Tailored Care and Support

Participants suggested that postoperative support should be individualized and that check-ups should be offered for a long time, some suggested lifelong, depending on individual needs. Participants desired more information and preparations before surgery, to be aware of the necessary lifestyle changes after surgery and the risk of weight regain. Psychological support was emphasized.

I think that you should focus more on working with people’s mind before cutting them open, that’s my opinion. I think the surgery is done a bit too easily. No. 7

Several participants requested more external control, thinking that regular check-ups would facilitate healthy eating, as well as providing an opportunity to detect substance or alcohol abuse, dysfunctional eating behaviors, or mental or somatic problems.

I wish that there was follow-up visits every year to stop any weight regain as soon as you notice an increase in weight. Then you can maybe stop it at 120 or 130 kg and advice “you have to do something”, instead of allowing it to continue... No. 2

Some wanted individual contacts only, while others wished for group support with peers who had undergone surgery to see how different people managed life after surgery.

So I think a group [of peers] would have been better, to attain a feeling of ‘I am not alone in this shameful situation of having gained weight, but we are many in the same boat’ and we could get help from a dietitian then, while being honest with each other, the benefits of being in a group. No. 5

Additionally, it was proposed that participants’ close relations should receive information, and in some cases, marriage counseling, to enable a supporting family environment.

.. it would be very important, for the first year, that you have enormous support at home. I would like to have practical tips, like you have to help each other with housework, with kids and everything so you can sit down and eat your meal in peace and quiet. There needs to be time to prepare meals. That’s why it is important that your partner also invest time. No. 8

One participant felt support had been even more than enough. Her problems with weight regain had been taken seriously, resulting in first a thorough physical examination and then a referral to the obesity clinic.

I received a lot of dietary support and they were on me all the time and tried to get me to come there [to the clinic]. It was I who were not really…too keen on it. No. 10

## Discussion

### Main Findings

In this qualitative interview study of patients with post-bariatric surgery weight regain, two overall themes were identified; *A lonely struggle* and *Others as sources of compassion and control*. For most participants, support during the postoperative weight regain period was either absent, not extensive enough, confusing, or even discouraging. Nonetheless, several interviewees had also experienced and exemplified supportive actions and empathetic response from family, friends, peers, and healthcare providers.

### Insufficient Support

Participants were critical towards both the extent and content of their perceived support. Their desire for individualized follow-up agrees well with previous findings about the need for psychological support, peer- and nutritional support [16, 17]. National guidelines for follow-up [18] were lacking during the time of surgery for the participants. The current Nordic guidelines for follow-up after obesity surgery suggest at least annual, life-long follow-up with laboratory-assessed nutritional status, as well as assessment of postoperative adverse events [18].

Sharman et al. suggest that the need for follow-up is higher during the first postoperative year [16]. Others claim that the need for support increases later on, when follow-up visits are offered less frequently [19]. Similar differences of opinion appeared in this study population.

### Information Barriers

Several participants were unaware of weight regain risks and lacked tools to maintain or alter eating habits when the surgical intervention itself no longer seemed to regulate the food intake. They did not know where to seek help when weight regain occurred. Though information may have been given, some participants had found it difficult to read brochures or remember information, or felt they were unreceptive at the time. This may indicate that the extent of health literacy, defined as “the degree to which individuals can obtain, understand, and communicate about health-related information needed to make informed health decisions” [20] should be considered by healthcare providers before surgery. The majority of participants also had physical comorbidities, psychiatric conditions, accidents, or other subversive life events that required attention, which may further explain why efforts to maintain a healthy lifestyle could have been less prioritized.

### Shame, Self-Blame, and Dysfunctional Coping Strategies

Although causal effects cannot be concluded from this exploratory study, participants themselves suggested that lack of, or unfavorable, support may have worsened their weight development. Their feelings of abandonment and self-accusations were aggravated by negative comments or misunderstandings. The psychological and emotional stress may have induced comfort eating, or deflated motivation to engage in lifestyle changes.

Participants were exposed to stigmatizing treatment, which is known to have a negative impact on mental health and self-esteem [21], and may amplify dysfunctional eating behaviors. Feelings of shame and guilt among persons with weight regain have been reported previously [22, 23]. Persons with weight regain may feel more stigma, because the shame of becoming obese and needing bariatric surgery, and subsequently the failure of postoperative weight loss maintenance [24].

Participants used social isolation and avoidance to minimize the risk of being judged by others. Consequently, they simultaneously deprived themselves from the possibility to get support from family and friends, and may have delayed getting professional help. Due to the presence of shame and weight stigma, education for healthcare providers may be needed to increase obesity knowledge and counteract stigmatization in order to minimize negative impact on patients [25]. In addition, informing patients about the possibility of weight regain in a non-stigmatizing way, stressing both the fact that weight regain is a treatment failure (and not a personal failure) and that additional medical treatments exists, could reduce shame and lower the threshold for seeking professional help.

### Empowering Support

According to the Social Learning Theory [26], a higher degree of perceived internal locus of control is associated with greater success in weight management programs [27]. Participants in the present study commonly blamed themselves for weight loss failure, while also expressing a recurrent wish to have others take on responsibility and exert control. The same was described in a study by Ogden et al. [28] where bariatric surgery was perceived as the ultimate tool for external control, while successful bariatric surgery in turn seemed to improve patients’ sense of control over their eating behaviors, suggesting a bidirectional association. Previous negative experiences of weight management and a perceived lack of favorable support may have contributed to patients’ dysfunctional coping responses to weight regain.

Self-efficacy is necessary for behavior changes, and the Social Cognitive Theory displays a complex interaction between the environment, the individual, and the behavior itself called reciprocal determinism [29]. More successful weight loss after bariatric surgery has been associated with greater self-efficacy [30], as have positive support from peers and family [31]. Participants’ lack of support as well as misunderstandings and negative remarks may thus have influenced and reduced their self-efficacy. This emphasizes social support as an important factor for maintaining weight loss after bariatric surgery. In our study, support from family members was highlighted, but the way it was executed was not always favorable. As family function and marital dynamic may be negatively affected by bariatric surgery [32], the present study is in line with previous research suggesting that counseling, or education, could be offered to patients’ significant others. Patients’ need of support may be more easily met, if friends and family are aware of the massive changes in eating habits and physical activity that are required after bariatric surgery.

### Strengths and Limitations

For this study’s trustworthiness and credibility [33], participants were recruited by purposeful sampling. Thus, they had relevant and various experiences about weight regain after bariatric surgery, which enabled a deep understanding. Due to the diversity of the sample, transferability or generalizability to similar contexts was strengthened [33]. The sample varied in gender, age, and country of origin, and the proportion of males/females mirrors the population that undergoes bariatric surgery in Sweden [34].

The credibility of the findings was further strengthened by the authors’ long engagement in the field of obesity, the researcher triangulation during the analysis phase, and the collaboration with co-authors that facilitated reflection and reduced the risk of bias from the first authors’ (LT) preconceptions [11]. Illustrating quotes further increased dependability.

A limitation is that only treatment-seeking persons were interviewed. Thus, it might not be generalized to other groups. Data from other participants, for example, those without weight regain, may have rendered other narratives about support. However, our data contained descriptions of both beneficial and discouraging treatment.

Another limitation is that only persons who had undergone gastric bypass were included. However, only 15% of the total bariatric surgery population in Sweden have undergone sleeve gastrectomy [35]. Additionally, most sleeve operations were performed within the last 5 years, leaving less time for weight regain to occur. Nonetheless, we do not believe that type of surgery is associated with the perceptions, feelings, experiences, and opinions about support post-surgery.

The retrospective perspective gave the participants the opportunity to look back and reflect upon the years after bariatric surgery. Although the surgical time frame was different for the different participants, increasing the likelihood that details were forgotten for those whose bariatric surgery was conducted a long time ago, the purpose was to explore subjective experiences and perceptions and such narratives can still be considered credible [36].

## Conclusions

For some patients, existing postoperative care is insufficient to avoid weight regain after bariatric surgery. Patients view weight regain as a personal failure, and shame and self-blame may make them reluctant to seek professional help or social support. Therefore, an empathetic and non-judgmental approach was desired. This study further suggests that regular follow-ups may be crucial, and multidisciplinary support is needed to target difficulties that may impact weight-management after bariatric surgery. Specific support groups for those with weight regain may be beneficial. Additionally, offering patients’ family members education about bariatric surgery may enable a more supportive home environment.
